# Fast neutron-induced structural rearrangements at a soybean *NAP1* locus result in *gnarled* trichomes

**DOI:** 10.1007/s00122-016-2735-x

**Published:** 2016-06-09

**Authors:** Benjamin W. Campbell, Anna N. Hofstad, Suma Sreekanta, Fengli Fu, Thomas J. Y. Kono, Jamie A. O’Rourke, Carroll P. Vance, Gary J. Muehlbauer, Robert M. Stupar

**Affiliations:** 1 Department of Agronomy and Plant Genetics, University of Minnesota, Saint Paul, MN 55108 USA; 2 USDA-ARS, Corn Insects and Crop Genetics Research, Iowa State University, Ames, IA 50011 USA; 3 Department of Plant Biology, University of Minnesota, Saint Paul, MN 55108 USA

## Abstract

**Key message:**

**Three adjacent and distinct sequence rearrangements were identified at a NAP1 locus in a soybean mutant. Genetic dissection and validation revealed the function of this gene in soybean trichome development.**

**Abstract:**

A soybean (*Glycine max* (L.) Merr.) *gnarled* trichome mutant, exhibiting stunted trichomes compared to wild-type, was identified in a fast neutron mutant population. Genetic mapping using whole genome sequencing-based bulked segregant analysis identified a 26.6 megabase interval on chromosome 20 that co-segregated with the phenotype. Comparative genomic hybridization analysis of the mutant indicated that the chromosome 20 interval included a small structural variant within the coding region of a soybean ortholog (Glyma.20G019300) of Arabidopsis *Nck*-*Associated Protein 1* (*NAP1*), a regulator of actin nucleation during trichome morphogenesis. Sequence analysis of the candidate allele revealed multiple rearrangements within the coding region, including two deletions (approximately 1–2 kb each), a translocation, and an inversion. Further analyses revealed that the mutant allele perfectly co-segregated with the phenotype, and a wild-type soybean *NAP1* transgene functionally complemented an Arabidopsis *nap1* mutant. In addition, mapping and exon sequencing of *NAP1* in a spontaneous soybean *gnarled* trichome mutant (T31) identified a frame shift mutation resulting in a truncation of the coding region. These data indicate that the soybean *NAP1* gene is essential for proper trichome development and show the utility of the soybean fast neutron population for forward genetic approaches for identifying genes.

**Electronic supplementary material:**

The online version of this article (doi:10.1007/s00122-016-2735-x) contains supplementary material, which is available to authorized users.

## Introduction

The plant trichome is an elongated epidermal cell that undergoes cell enlargement away from the plant surface. Trichomes develop on the surfaces of leaves, stems, petioles, and some reproductive organs (Vermeer and Peterson 1979; Nyman 1993). Trichomes perform many biological functions, including plant defense against insect predation, where they can affect larval growth and insect preferences (Levin 1973; Robbins et al. 1979; Hulburt et al. 2004), and adaptation of desert plants to drought conditions by increasing the leaf reflectance, which helps to moderate leaf temperatures without requiring increased transpiration (Ehleringer and Mooney 1978). Economically, cotton seed trichomes compose the fibers that make cotton a valuable commodity. Thus, an understanding of the genetic control of trichome development impacts both agricultural and economic productivity.

There has been considerable interest in understanding the genetic controls that underlie trichome formation, particularly in model plant systems. In Arabidopsis, several genes required for trichome formation have been characterized. *GLABRA1* (*GL1*) and *TRANSPARENT TESTA GLABRA1* (*TTG1*) are important for trichome initiation (Oppenheimer et al. 1991; Walker et al. 1999). *GLABRA2* (*GL2*) controls normal trichome morphogenesis (Rerie et al. 1994), and *TRIPTYCHON* (*TRY*) and *CAPRICE* control the spacing pattern of trichomes across the leaf surface (Schellmann et al. 2002). *ZWICHEL* (*ZWI*), *CONSTITUTIVE PATHOGENE RESPONSE5 (CPR5)*, *TRANSPARENT TESTA GLABRA2* (*TTG2*), and *KAKTUS (KAK)* affect trichome branching (Oppenheimer et al. 1997; Kirik et al. 2001; Johnson 2002; El Refy et al. 2003; Downes et al. 2003), and mutations in *CROOKED* (*CRK*) and *GNARLED* (*GRL*) cause distorted trichomes (Mathur et al. 2003; Deeks et al. 2004; El-Assal et al. 2004). However, to our knowledge, the only previously isolated soybean gene affecting any trichome trait is the T allele, which has pleiotropic effects on the color of the trichome, hilum, and seed-coat (Woodworth 1921; Zabala and Vodkin 2003).

Several soybean mutagenesis platforms have been developed for functional characterization of soybean genes (reviewed by Campbell and Stupar 2016). These platforms include chemical mutagenesis (Cooper et al. 2008; Gillman et al. 2014), transposon tagging (Palmer et al. 2008a, b; Mathieu et al. 2009; Hancock et al. 2011; Cui et al. 2013; Raval et al. 2013), and irradiation mutagenesis (Men et al. 2002; Bolon et al. 2011; Gillman et al. 2014). Chemical mutagenesis causes single nucleotide polymorphisms (SNPs), and transposon tagging results in insertion mutants (Cooper et al. 2008; Palmer et al. 2008a, b; Mathieu et al. 2009; Hancock et al. 2011; Cui et al. 2013; Raval et al. 2013; Gillman et al. 2014). In contrast to the limited mutation types caused by chemical and transposon mutagenesis, irradiation mutagenesis has been reported to induce a wide variety of mutation types, including structural rearrangements (e.g. deletions, duplications, translocations, and inversions) of varying sizes, and SNPs (Bolon et al. 2011, 2014; Belfield et al. 2012).

Previously, forward genetic approaches to identify causative mutations induced through mutagenesis were often slow and typically required initial coarse mapping followed by one or more rounds of fine-mapping to positively identify a causative variant. Genetic methods, such as bulked segregant analysis (BSA) (Michelmore et al. 1991), have been developed to facilitate the coarse mapping of qualitative traits; however, fine-mapping based on the phenotyping and genotyping of individuals from large populations is arduous. The advent of new molecular technologies has rapidly decreased the time required to physically and genetically map potential causative polymorphisms. For example, irradiation-induced mutations can be detected using array Comparative Genomic Hybridization (aCGH) or genome resequencing. The aCGH approach is useful for quickly identifying sufficiently large (≥2 kb) deletions and duplications, as has been demonstrated in a variety of plant species such as *Arabidopsis thaliana* (Gong et al. 2004), clementine (*Citrus clementina* Hort. Ex Tan. Cv. Clemenules) (Ríos et al. 2008), rice (*Oryza sativa*) (Bart et al. 2010), and soybean (Bolon et al. 2011, 2014). Furthermore, sequencing-based genotyping of BSA samples can be used to decrease the time required to identify chromosomal loci that co-segregate with qualitative traits. This concept has been demonstrated using a range of different sequencing approaches (including sequencing of RNA, whole-genomes, and exomes) and has been demonstrated in several plant species, such as tomato *Solanum lycopersicum* (Illa-Berenguer et al. 2015), maize (Liu et al. 2012; Haase et al. 2015), rice (Takagi et al. 2013; Yang et al. 2013), barley (Mascher et al. 2014), and Arabidopsis (James et al. 2013; Zhang et al. 2014).

We were intrigued to test whether a combination of aCGH and whole genome sequencing-based bulked segregant analysis (WGS-BSA) could facilitate the rapid cloning of the causative gene(s) from an irradiated soybean mutant. In this study, we report the identification of a causative mutation underlying a previously identified soybean fast neutron mutant that exhibits *gnarled* trichomes. A combination of aCGH and WGS-BSA was used to identify a *Nck*-*Associated Protein 1* (*NAP1*) candidate gene for this trait, and subsequent genetic and molecular analyses confirmed the essential function of this gene in trichome development.

## Materials and methods

### Populations and phenotyping

A *gnarled* trichome mutant, R55C01 (Soybase.org mutant FN0175501), was identified in a soybean fast neutron mutant population developed at the University of Minnesota using the soybean line ‘M92-220’ which was derived from the variety ‘MN1302’ (Orf and Denny 2004; Bolon et al. 2011). This mutant was crossed to the wild-type accession ‘Noir 1’ [subline Noir 1-SGC-01 (McHale et al. 2012)] to generate a segregating mapping population. The F_1_ hybrid and the subsequent segregating F_2_ and F_3_ individuals were grown in the greenhouse and visually phenotyped.

### Detection of structural variants using comparative genomic hybridization microarrays

The aCGH array was designed using the first version of the soybean reference cv. ‘Williams 82’ genome sequence Glyma.Wm82.a1.v1.1 (Bernard and Cremeens 1988; Schmutz et al. 2010). The array was composed of unique sequence probes (50–60mers) spaced across the genome at an interval typically ranging from 0.5 to 1.1 kb. The methods used for the labeling and the aCGH analysis were conducted according to the methods described in previous studies (Haun et al. 2011; Bolon et al. 2011, 2014; Anderson et al. 2014), using ‘M92-220’ as the reference sample for the array. Genomic DNA was isolated from leaf tissue using the Qiagen DNeasy kit, and 500 ng of genome DNA from each line was used for the labeling reaction. The mutant DNA was labeled using Cy3 dye and ‘M92-220’ reference sample was labeled with Cy5 dye. The labels were incorporated using the 3′–5′ exo-Klenow fragment from DNA polymerase I. The labeled DNA was quantified and hybridized for 72 h at 42° C to the 700k feature NimbleGen aCGH array. The methods used for array scanning and data analyses have been previously described (Bolon et al. 2011).

### Sequencing of R55C01, ‘Noir 1’, and F_2_ bulks

Fifty F_2_ individuals with wild-type trichomes and 50 F_2_ individuals with mutant trichomes were chosen from the ‘Noir 1’ × R55C01 population to compose the two mapping bulks. Genomic DNA of both bulks, the mutant R55C01, and the wild-type ‘Noir 1’ individual was extracted from leaf tissue using a Qiagen DNeasy kit. DNA samples were submitted to the University of Minnesota Genomics Center (UMGC) for sequencing on an Illumina HiSeq 2000 producing 101 bp paired-end reads with the goal of achieving an average sequencing coverage of 30×. Scythe (https://github.com/vsbuffalo/scythe) was used to remove adapter sequences from the 3′ ends of reads, with a 5 % prior on contamination rate. Sickle (https://github.com/najoshi/sickle) was then used to remove bases with a Phred quality below 20. Cleaned reads were aligned to the updated soybean reference genome assembly Glyma.Wm82.a2.v1 (Song et al. 2016) using BWA-MEM version 0.7.5a (Li 2013). Mismatch penalties and alignment reporting parameters were adjusted to report alignments with approximately 1 % mismatch from the reference. Alignments were sorted, de-duplicated, and labeled with read groups using Picard Tools version 1.107 (http://broadinstitute.github.io/picard/). Alignments were then re-aligned around potential insertion/deletion polymorphisms using the Genome Analysis Tool Kit (GATK) version 3.1-1. To minimize computational time while obtaining genome-wide coverage, we called SNP variants at the genomic positions previously identified for the SoySNP50K genotyping platform (Song et al. 2013) rather than all possible SNPs. Variants were called using the GATK UnifiedGenotyper (McKenna et al. 2010; DePristo et al. 2011; Van der Auwera et al. 2013). A custom Python script called VCF_MAF.py (available at https://github.com/TomJKono/Misc_Utils) was then used to estimate allele frequencies and calculate read depths in each bulk.

### Whole genome sequencing-based bulked segregant analysis (WGS-BSA)

The bulk allele frequencies were initially calculated as the Glyma.Wm82.a2.v1 reference or the alternate state using custom PERL script for the SoySNP50K positions (Song et al. 2013, 2016). To utilize these data for mapping, the allele frequencies were converted to allele frequencies of the wild-type ‘Noir 1’ parent at each position in both bulks based on the alternate or reference allele state of ‘Noir 1’ at each SNP position from the ‘Noir 1’ sequence data. Non-polymorphic SNPs (having allele frequencies in both bulks of greater than or equal to 0.9 or less than or equal to 0.1), SNPs with missing data in either bulk, and SNPs with read counts less than ten in either bulk were removed from the dataset. The allele frequencies were plotted and graphically analyzed for spreads in allele frequency.

### Genotyping and phenotyping of segregating F_3_ individuals

R55C01, ‘Noir 1’, and F_3_ families derived from different F_2_ individuals were planted in the greenhouse and visually phenotyped for the presence of wild-type or *gnarled* trichomes. Genomic DNA was extracted from a single F_3_ individual from each homozygous family and from one mutant and one wild-type plant from each segregating F_3_ family. The genomic sequence of R55C01 was utilized to design PCR primers (Supplemental Table 1) that amplified distinct amplicons for the wild-type and mutant alleles.

### Validation of chromosome rearrangements in *GmNAP1*

PCR was used to validate the chromosome rearrangements identified by whole genome sequencing at the locus of the candidate gene, *Glycine max NAP1* (*GmNAP1*). PCR primers were designed using the genomic sequence of R55C01 (Supplemental Table 2).

### RNA sequencing of R55C01 and ‘M92-220’ root, seed, and leaf tissue

R55C01 and ‘M92-220’ seeds were imbibed in sterile water for 48 h before being transferred to pots containing quartz sand, with four seedlings planted per pot and later thinned to one plant. Plants were placed in a growth chamber at 28 °C, oscillating between 16 h of light and 8 h of dark. Each pot was watered daily with 500 mL of nutrient solution (O’Rourke et al. 2014). At the V2 stage, emerging trifoliates and total root tissues were harvested and immediately immersed in liquid nitrogen from three biological replicates. Developing seeds were harvested at seed stage 0 (10 mg) from three additional biological replicates. RNA was extracted from leaf, root, and developing seeds using the Qiagen RNeasy kit and submitted to the UMGC, where samples were sequenced on an Illumina HiSeq2000. Illumina library preparation, clustering and sequencing reagents were used throughout the process, following the manufacturer’s recommendations. Samples were sequenced as 50 bp paired-end reads with an insert size of 200 bp. On average, each sample generated 26 million paired-end reads. Read quality was confirmed using FASTQC (www.bioinformatics.babraham.ac.uk/projects/fastqc/). Reads were aligned to the reference genome version Glyma.Wm82.a1.v1 (Schmutz et al. 2010) using TopHat2 (Kim et al. 2013). Reads mapping to genic regions were identified using a combination of SAMtools and HTseq (Li et al. 2009; Anders et al. 2014). Differential gene expression and exon expression analyses were performed in R (R Development Core Team 2006) using DESeq (Anders and Huber 2010) and EdgeR (Robinson et al. 2010), respectively.

### Mapping the *p2* introgression interval

The similarity of the R55C01 trichome mutant phenotype to the phenotypic descriptions and images of the *p2* trichomes of line T31 (PI548159) suggested that the *p2* allele was caused by a mutation affecting the same gene or pathway as the R55C01 mutant (Stewart and Wentz 1926; Bernard and Singh 1969; Singh et al. 1971; Healy et al. 2005). Bernard et al. (1991) backcrossed the *p2* allele into the cv. ‘Harosoy’ (PI548573) (Weiss and Stevenson 1955) and cv. ‘Clark’ (PI548533) (Johnson 1958) backgrounds to generate two ‘Harosoy’ *p2* backcross lines (PI547713 and PI547743) and three ‘Clark’ *p2* backcross lines (PI547449, PI547565, and PI547566). The SoySNP50K data for the three parents and five *p2* backcross lines (Song et al. 2015) were obtained from SoyBase (http://soybase.org). SNPs that were not polymorphic between mutant line T31 and wild-type lines ‘Clark’ and ‘Harosoy’ were removed. The genome was then scanned for SNPs at which T31 and all five *p2* backcross lines shared the same allele.

### Sequencing the *GmNAP1* gene in T31

Seed of T31 (PI548159) was obtained from USDA Germplasm Resources Information Network, and T31 genomic DNA was extracted from leaf tissue. The candidate gene for this study, Glyma.20G019300, has a length of 22.5 kb, not including the promoter sequence, and the predicted transcript length is 4.8 kb (Song et al. 2016). PCR primers were designed to amplify the 5′UTR, 3′UTR, and all 23 exons including all splice site junctions (Supplemental Table 3). Reactions were PCR purified using a QIAquick PCR Purification Kit (Qiagen) and sequenced at the UMGC. Sequences were aligned to the sequence of gene model Glyma.20G019300 based on the reference sequence Glyma.Wm82.a2.v1.1 (Song et al. 2016).

### Transgenic complementation of Arabidopsis *glr*-*4* using *GmNAP1*

The *GmNAP1* construct was designed in a pMDC123 backbone and consisted of the native promoter amplified from ‘Williams 82’ driving the synthesized *GmNAP1* cDNA (Piscataway, NJ) followed by a NOS terminator. The pMDC123 vector used also contained a 35S promoter driving a BAR herbicide resistance gene.

The Arabidopsis *nap1* mutant, *grl*-*4* (El-Assal et al. 2004), was transformed with the *GmNAP1* construct using the floral dip method (Clough and Bent 1998), and T_1_ seeds were planted in a flat containing standard potting mix. Ten and seventeen days after germination the flat was sprayed with a 0.01 % solution of glufosinate and resistant plants were then transferred to individual pots. Leaf tissue was collected from each T_1_ individual. PCR primers that amplified across the junction between the promoter and first exon of Glyma.20G019300 were used to test for the presence of *GmNAP1* construct in ‘Williams 82’, *grl*-*4*, and 20 T_1_ individuals with wild-type trichomes (Supplemental Table 4).

### Data availability

The aCGH data for mutant R55C01 (also known as FN0175501) can be found in the National Center for Biotechnology Information (NCBI) Gene Expression Omnibus (https://www.ncbi.nlm.nih.gov/gds) accession number GSM1402716. All DNA and RNA sequence data can be found in the NCBI Sequence Read Archive (http://www.ncbi.nlm.nih.gov/sra/) repository. Whole genome DNA sequence data for R55C01 and M92-220 can be found in SRP036841 (accession numbers SRX467193 and SRX467183, respectively). The RNA-seq data for R55C01 and M92-220 can be found in SRP074365 (accession numbers SRX1742547-SRX1742555 and SRX1742565-SRX1742573, respectively.)

## Results

### Identification and mapping of the *gnarled* trichome mutant

**Fig. 1:**
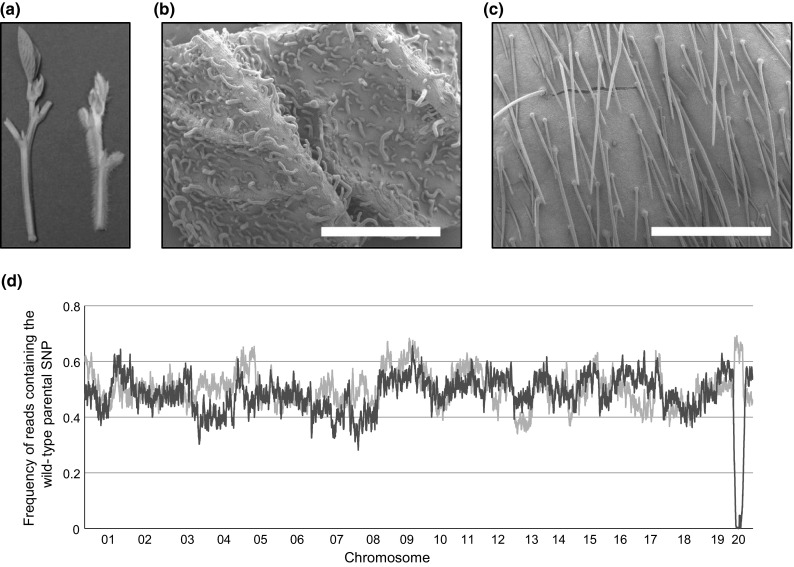
Phenotype and coarse genetic mapping of the *gnarled* trichome mutant. **a** Phenotypes of mutant (*left*) and wild-type (*right*) shoots. SEM leaf trichome images of the *gnarled* mutant R55C01 **(b)** and the wild-type line ‘M92-220’ **(c)**. The mutant trichomes are wide, short, flaccid, and lay on the surface of the leaf or stem, as compared to the wild-type trichomes (narrow, long, straight, and project outward from the leaf or stem). *Scale bars* in **b** and **c** are 1 mm. **d** BSA-WGS allele frequencies were calculated for F_2_ bulked samples that consisted of 50 mutant (*red lines*) and 50 wild-type (*blue line*) individuals. The allele frequencies were calculated as the proportion of reads containing the wild-type parental SNP (i.e. SNPs that match the wild-type parent ‘Noir 1’) at each position for over 16,000 polymorphic SoySNP50K positions. The allelic frequencies are shown as the average value across a 21 SNP sliding window. The obvious spread in allele frequencies indicates that the causative locus is located on chromosome 20 (color figure online)

Several morphological and developmental mutants were discovered during the visual phenotypic screening of the soybean fast neutron population generated at the University of Minnesota (Bolon et al. 2011). Mutant R55C01 (Soybase mutant FN0175501) was identified as a short trichome mutant (SOY:0001804) (Fig. 1a). Scanning electron microscope images of the leaves from wild-type and mutant plants indicated that the mutant has a *gnarled* trichome phenotype (Fig. 1b, c), which is characterized by trichomes that are swollen, twisted and reduced in length (Szymanski et al. 1999; Deeks et al. 2004; El-Assal et al. 2004). The *gnarled* mutant trichomes are shorter, exhibit wide, flaccid shafts (SOY:0001720), lay on the surface of the leaf or stem (SOY:0001977), and have round, blunt tips (SOY:0001722).

aCGH was conducted on an M5 mutant plant to identify potential causative mutations [this plant was assigned the identification number FN0175501.x2.02.01.M5 in Bolon et al. (2011)]. The aCGH results did not identify any duplications but did identify two deletions in the mutant genome: a putative 26 kb deletion on chromosome 5 and a putative ~2 kb deletion on chromosome 20. However, the aCGH method is not sensitive enough to detect some types of rearrangements (inversions and translocations), small deletions, and small duplications, which may underlie the mutant phenotype.

Genetic mapping was conducted to identify the genomic interval co-segregating with the trichome phenotype. The mutant was outcrossed to the wild-type accession ‘Noir 1’ to generate the mapping population. The F_1_ plant had wild-type trichomes, and the F_2_ population segregated in a 3:1 wild-type to mutant ratio (144 to 53; Chi-squared *p* value = 0.537 for one locus), indicating that the trichome mutant phenotype was recessive and was caused by a mutation at a single locus.

WGS-BSA was conducted using a bulked sample of F_2_ mutant segregants and a bulked sample of F_2_ wild-type segregants. The allele frequencies were calculated with respect to the ‘Noir 1’ SNP state such that SNPs closely linked to the causative locus would exhibit a mutant bulk allele frequency of approximately zero and a wild-type bulk frequency of approximately 0.66 (as two out of every three wild-type plants would be expect to be heterozygous at the causative locus). The allele frequencies at each SNP position were visualized as the proportion of reads derived from ‘Noir 1’ and averaged across a 21 SNP sliding window (Fig. 1d). Chromosomes 1 through 19 did not show any major divergence in allele frequencies between the bulks. However, the chromosome 20 allele frequencies exhibited the expected divergence for the causative locus (Fig. 1d).

**Fig. 2:**
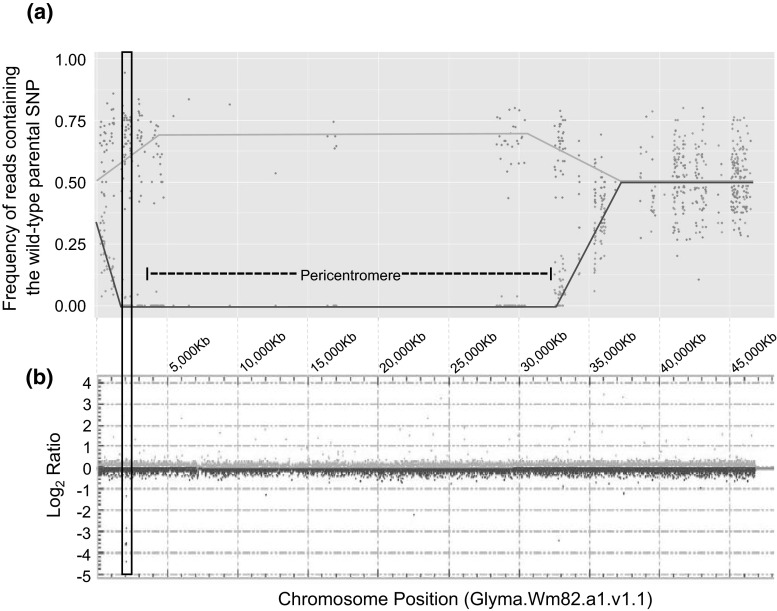
Genetic mapping of the *gnarled* mutant and physical mapping of the deletion on chromosome 20. Coincidental mapping of the **a** WGS-BSA mapping interval with **b** a deletion detected by array Comparative Genomic Hybridization (aCGH). In **a**, blue data points indicate the ‘Noir 1’ SNP frequency at each marker position in the wild-type bulk; red data points indicate the ‘Noir 1’ SNP frequency at each marker position in the mutant bulk. *Blue* and *red lines*, respectively, are drawn in **a** to assist in visualizing the separation in the bulk allele frequencies. A *vertical rectangle* shared between **a** and **b** identifies the chromosome region containing the *GmNAP1* gene. In **b**, probes below the 0.0 log_2_ value indicates the absence of mutant DNA (i.e. a putative deleted segment) (color figure online)

A detailed analysis of chromosome 20 showed that the mutant bulk had an average ‘Noir 1’ allele frequency of zero for a 26.6 Mb interval spanning the pericentromere on chromosome 20, between positions 1.67 and 28.3 Mb (Fig. 2a). This 26.6 Mb interval only encompasses 65 gene models, as the region is mostly heterochromatic. The mutant bulk’s average ‘Noir 1’ allele frequency of zero indicates that the mutant parent markers in the 26.6 Mb interval co-segregated with the F_2_ mutant phenotype. For the same interval, the wild-type bulk had the excepted average ‘Noir 1’ allele frequency of 0.66. The 26.6 Mb interval overlapped with one structural variant detected by the aCGH experiment, the approximately 2 kb deletion (Fig. 2b). This deletion, presumably generated by the fast neutron mutagenesis, was located within a single gene model, Glyma.20G019300. The nearest ortholog to this gene in Arabidopsis is *NAP1*, or *Nck*-*Associated Protein 1* (gene model AT2G35110), and is involved in the actin cytoskeleton formation (Deeks et al. 2004; El-Assal et al. 2004). The soybean gene model Glyma.20G019300 (named Glyma20g02370 in previous genome releases) has high amino acid similarity to this Arabidopsis *NAP1* ortholog (87.4 %). No other Arabidopsis gene model showed high sequence similarity to this soybean gene. Furthermore, the Arabidopsis *nap1* trichome mutant (*grl*-*4*) has a phenotype with swollen, twisted, and shorter trichomes, similar to the soybean mutant phenotype observed in R55C01 (Deeks et al. 2004; El-Assal et al. 2004).

To validate the mapping results, co-segregation between the trichome phenotype and the candidate mutation was tested on segregating F_3_ progeny. A co-dominant PCR marker was designed using three primers to amplify unique bands for the mutant and wild-type alleles (Supplemental Table 1, Supplemental Fig. 1A). The phenotypes of 50 F_3_ individuals, representing different F_2:3_ families, perfectly co-segregated with their expected genotypic classes (mutant: mt/mt or wild-type: wt/wt or wt/mt) (Supplemental Fig. 1). Altogether, these data and prior information about the *NAP1* gene indicated that Glyma.20G019300 was the leading candidate gene for the soybean *gnarled* phenotype.

### Complex rearrangements detected in Glyma.20G019300

**Fig. 3:**
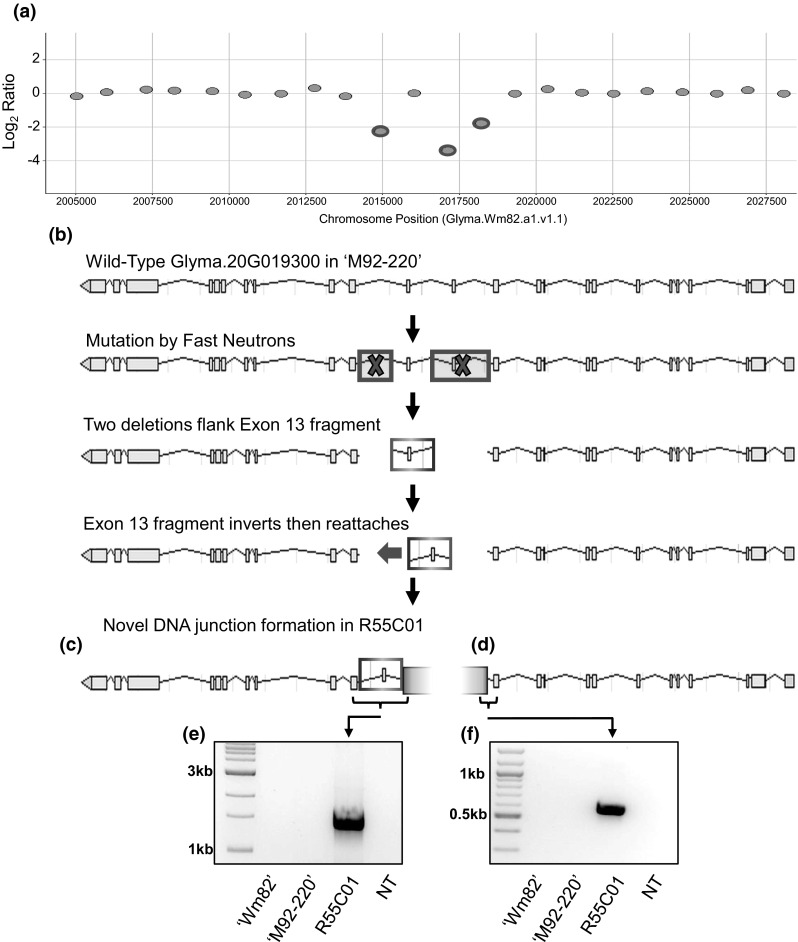
Mutations in the candidate gene demonstrate the complexity of mutations that can occur by fast neutron mutagenesis. **a** aCGH report depicting two deletions in *GmNAP1* indicated by probes with corrected log_2_ ratios of less than −2. aCGH array was designed using version 1 assembly (Glyma.Wm82.a1.v1.1), thus v1 positions are listed. **b** Wild-type Glyma.20G019300 and fast neutron mutations to the gene. **c** The inverted 13th exon connected to the second half of the gene forms a novel junction between Gm20:2,010,290 and Gm20:2,007,928 (positions are according to the version 2 genome assembly, Glyma.Wm82.a2.v1). The Gm20:2,009,152 side of the inverted fragment is connected to sequence found at Gm20:16,920,485. **d** The first half of the gene is interrupted at Gm20:2,012,311 and is connected to sequence found at Gm20:16,939,673. A novel 22 bp sequence was found in the junction. PCR amplification was used to confirm the novel DNA junctions created by fast neutron mutagenesis. **e** A 1.4 kb fragment spanning across two novel junctions created in the second half of the gene. **f** A 605 bp fragment spanning the novel junction created in the first half of the gene. For **e** and **f**, the samples tested were (*left* to *right*): ‘Williams 82’, ‘M92-220’, R55C01, and a no template control. The orientations of the sequences at the junctions do not suggest that a single contiguous piece was inserted into Glyma.20G019300, and the extent of chromosomal rearrangements that occurred on chromosome 20 is unclear at this time

The aCGH data detected an approximately 2 kb deletion in the Glyma.20G019300 candidate gene, but was not able to resolve the fine structure of this event. Therefore, whole genome resequencing was conducted to resolve the specific breakpoints of this deletion. Surprisingly, the resequencing data revealed a much more complex structure to this locus than anticipated (Fig. 3).

Glyma.20G019300 consists of 23 exons stretched across 22,550 nucleotides (including exons, introns, and untranslated regions) that encode a protein with 1388 amino acids. Sequencing of the mutated allele resolved two distinct deletions (2021 and 1224 bp, respectively) nearby one another. The 1224 bp deletion was not originally detected by aCGH due to the deletion of only a single probe, but occurred upstream of the aCGH identified mutation. In addition, a 1138 bp segment separating these deletions was found in an inverted orientation. This inverted fragment consisted of the thirteenth exon and part of the twelfth and thirteenth introns. This fragment inverted and fused to a sequence 7 bp upstream of the fourteenth exon, forming a novel junction between positions Gm20:2,010,290 and Gm20:2,007,928. Finally, an unresolved chromosome rearrangement, possibly an intra-chromosomal translocation, was identified adjacent to the inversion–deletion junction. The Gm20:2,009,152 side of the inverted fragment was connected to sequence found nearly 15 Mb downstream in the reference genome, at Gm20:16,920,485. The first half of the gene was interrupted at Gm20:2,012,311 and was found to be connected to Gm20:16,939,673. A novel 22 bp sequence was also found in the junction. PCR primers were designed to span these junctions, and the amplicons confirmed the presence of the three novel junctions found by whole genome resequencing (Fig. 3).

The putative intra-chromosomal translocation remains unresolved. Orientation of the sequences at the junction Gm20:2,009,152 to Gm20:16,920,485 and the junction Gm20:2,012,311 to Gm20:16,939,673 do not suggest that a single contiguous piece was inserted into Glyma.20G019300. Rather, the orientations of the sequences suggest that additional chromosome rearrangements have likely occurred.

### RNA sequencing transcription analysis of the *gnarled* mutant

Complex rearrangements may alter transcription of the genes at and nearby the disrupted locus. To test this, RNA-seq analysis was conducted on leaf, seed, and root tissues from wild-type (‘M92-220’) and *gnarled* mutant individuals (R55C01). Approximately 95 % of the RNA-Seq reads were mapped to the soybean genome, and roughly 89 % of the read-pairs were mapped concordantly. Under normal conditions in wild-type plants, Glyma.20G019300 has been observed to be transcribed in all previously examined tissues (Libault et al. 2010; Severin et al. 2010a). Similarly, this gene was observed to be transcribed in all three tissues tested in this study; however, the *gnarled* mutant exhibited altered relative transcript abundances among the 23 exons (Supplemental Fig. 2).

Exons one through ten all exhibited similar transcription levels in mutant and wild-type plants, with some minor exon-specific fluctuations among the different tissues and genotypes, while exon 11 appeared to show higher relative transcription levels in the mutant (Supplemental Fig. 2). Exons 12, 13, and 14, however, exhibited essentially no transcription in the *gnarled* mutant (Supplemental Fig. 2), while the wild-type individuals exhibited transcription levels similar to the other exons of this gene. As described in the previous section, exon 12 resides within a deleted interval in the *gnarled* mutant, and an interval encompassing exon 13 is inverted relative to wild-type. These structural rearrangements may be expected to eliminate transcription in these intervals. Exon 14, while present and in proper orientation in the mutant, is directly adjacent to the exon 13 inversion and also appeared to be transcriptionally silent. The downstream exons, 15–23, exhibited fewer relative transcripts in the *gnarled* mutant relative to wild-type (Supplemental Fig. 2). Furthermore, it is worth noting that the transposition event that separates exons 11 and 13 in the *gnarled* mutant is of unknown size and sequence composition (Fig. 3). Therefore, it is possible that the reads observed from exons 1–11 and exons 15–23 are completely independent transcripts in the *gnarled* mutant line. Furthermore, gene model Glyma.07G221000, the nearest paralog to Glyma.20G019300, did not exhibit any exon-level transcript differences between the mutant and wild-type plants. This offers additional evidence that the transcriptional changes observed in Glyma.20G019300 are a consequence of the structural rearrangement per se, rather than RNA-interference or other post-transcriptional silencing mechanism.

Transcriptional alterations were also observed genome-wide between the *gnarled* mutant compared to wild-type, including 2299 genes differentially transcribed in at least one of the three tissue types. The genes differentially transcribed in leaf tissues included those involved in processes such as lipoxygenase activity (GO:0016165) and photosynthetic processes (GO:0009769 and GO:0016168), which are pathways that have been linked to trichome development (Schilmiller et al. 2010; Yan et al. 2012). However, homologs for genes previously demonstrated to be involved in trichome developmental processes, including monosaccharide and lignin biosynthesis (Marks et al. 2009), were not observed to be differentially transcribed between the *gnarled* mutant and wild-type in any tissue (data not shown). Among the 65 gene models located within the 26.6 Mb interval mapped by WGS-BSA, Glyma.20G019300 was the only differentially transcribed gene. None of the remaining 64 gene models exhibited differential transcription among any of the three tissue types.

### Complementation of *Atnap1* using *GmNAP1*

Due to the high similarity of both the *nap1* gene sequences and phenotypes, complementation of an Arabidopsis *nap1* mutant with *GmNAP1* could be used to validate the function of Glyma.20G019300. A construct consisting of 2 kb of the soybean *NAP1* promoter driving the soybean *NAP1* cDNA and a D35S promoter driving the BAR herbicide resistance gene was transformed into the Arabidopsis *nap1 gnarled* mutant (*grl*-*4*). Twenty T_1_ individuals were recovered that displayed wild-type trichomes, were resistant to glufosinate, and tested positive for the *GmNAP1* transgene based on PCR analyses (Supplemental Fig. 3). The functional complementation of the Arabidopsis *nap1* mutant indicates that *GmNAP1* is important for trichome formation and the two orthologs share functional homology.

### Identification of a spontaneous *NAP1* soybean mutant T31 (*p2*)

A search for historic soybean mutants with *gnarled* trichomes led to the identification of the mutant line T31 (PI548159) (Stewart and Wentz 1926; Bernard and Singh 1969; Singh et al. 1971; Healy et al. 2005). T31’s recessive *p2* mutant trichome allele was initially described as ‘puberulent’ but more closely resembles the *gnarled* phenotype. The *p2* allele was previously backcrossed into the cv. ‘Harosoy’ (PI548573) and into cv. ‘Clark’ (PI548533) to generate several advanced backcross lines (Weiss and Stevenson 1955; Johnson 1958; Bernard et al. 1991). There are two *p2* backcross (BC_6_) near-isogenic lines (PI547713 and PI547743) in ‘Harosoy’, and there are three *p2* backcross (one BC_6_ and two BC_7_) near-isogenic lines (PI547449, PI547565, and PI547566) in ‘Clark’ (Bernard et al. 1991). The SoySNP50K chip data (Song et al. 2015) obtained from SoyBase (http://soybase.org) were used to identify a genomic interval shared by T31 and the five *p2* backcross lines.

**Fig. 4:**
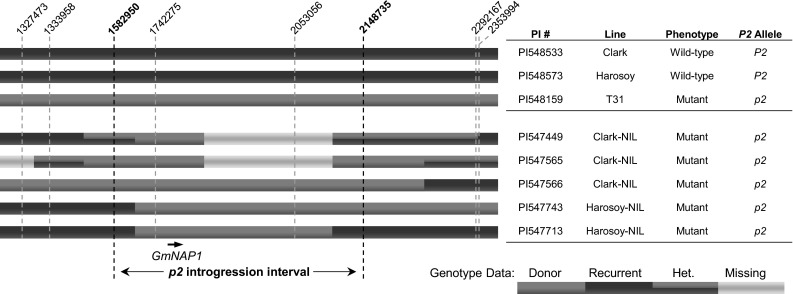
*p2* introgression interval identified on chromosome 20. Positions are given for polymorphic SoySNP50K markers in the genomic region. The *p2* allele, from the donor line T31, was previously backcrossed into the recurrent parents ‘Clark’ and ‘Harosoy’ to generate five Near Isogenic Lines (NILs) as part of the Soybean Isoline Collection. The recurrent parent genotypes are color coded in *blue*, and the donor parent genotypes are color coded in *red*, heterozygous (Het.) genotypes are color coded in *blue* and *red*, and missing genotypes are color coded in *gray*. Heterozygous scores are likely due to heterogeneity in the NIL. Examination of the five *p2* NILs’ genotypes identified a single introgression interval (566 kb) in the genome in which all five lines shared the donor parent genotype (Gm20:1,582,950–2,148,735). This interval contains the *GmNAP1* gene (Gm20:1,999,216–2,021,765) (color figure online)

A single genomic interval shared between T31 and the five *p2* backcross lines was located on chromosome 20 (Fig. 4). At position Gm20:1,742,275 (ss715636805), all five *p2* backcross lines carried the T31 allele, and at position Gm20:2,053,056 (ss715636914) three of the five *p2* backcross lines contained the T31 allele and two lines (PI547449 and PI547565) had missing genotypes. PI547565 had the T31 allele for the polymorphic SNP at Gm20:2,148,735 (ss71563945), adjacent to the missing genotype at Gm20:2,053,056, suggesting that the genotyping score at Gm20:2,053,056 would likely match T31. PI547449 had either missing data or heterozygous calls at all polymorphic SNP positions downstream of position Gm20:1,742,275 until Gm20:2,353,994 which had the ‘Clark’ allele. The observed heterozygous genotype calls are likely due to heterogeneity found between sampled individuals in a line rather than to residual heterozygosity within a specific individual of a line. The narrow 566 kb *p2* introgression interval, marked by the resumption of the recurrent parent haplotypes upstream at Gm20:1,582,950 (ss715636740) and downstream at Gm20:2,148,735 (ss715636945) contains the *GmNAP1* gene Glyma.20G019300. The inclusion of Glyma.20G019300 in the *p2* introgression interval and the similarity of the R55C01 and T31 trichome phenotypes suggested that *p2* could be caused by a mutation in Glyma.20G019300.

**Fig. 5:**
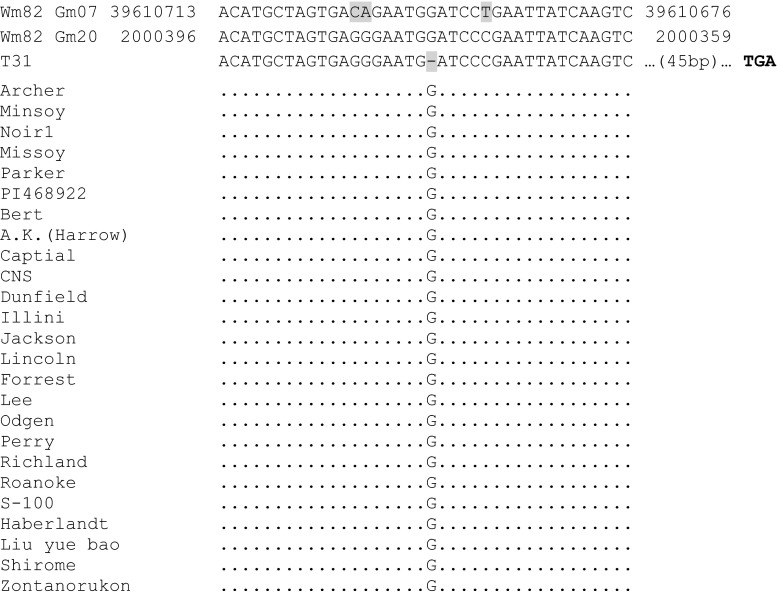
Sequence comparison of T31, ‘Williams 82’ (Wm82), and 25 diverse wild-type lines for the interval flanking the frame shift deletion found in T31. Sequencing of Glyma.20g019300 (*NAP1*) exon 22 in 25 diverse wild-type lines identified that the single base pair deletion is unique to T31. Sequence differences between the two soybean *NAP1* paralogs (highlighted in *gray*) make it possible to differentiate the chromosome 20 (Gm20) and chromosome 7 (Gm07) sequences. The given sequence positions are for genome assembly version 2 (Glyma.Wm82.a2.v1). T31’s 1 bp deletion is highlighted in* gray*. The resulting frame shift caused an early stop codon starting 64 bases downstream from the single base deletion. The early stop codon is shown in bold in the T31 downstream sequence

Sequencing the exons of Glyma.20G019300 from T31 identified a single base pair deletion in the 22nd exon (Fig. 5). The resulting frame shift mutation and early stop codon resulted in the mutation or loss of 202 amino acids (14.5 % of the gene). Sequence analysis of the locus in 25 wild-type diverse soybean accessions (McHale et al. 2012) confirmed that the single base pair deletion is unique to T31.

## Discussion

The present study combined aCGH and WGS-BSA methods to identify fast neutron-induced chromosome rearrangements in Glyma.20G019300 that caused a *gnarled* trichome phenotype in the soybean mutant R55C01. This gene is the soybean ortholog of the well-characterized Arabidopsis *NAP1* gene (Deeks et al. 2004; El-Assal et al. 2004). The morphological development of trichomes is guided by the actin cytoskeleton and thus proper actin nucleation is critical for proper trichome formation (Beilstein and Szymanski 2004). The *NAP1* gene was identified as a component of the SCAR/WAVE protein complex that activates the ARP2/3 complex involved in actin nucleation, and mutations in this gene have been shown to result in the *gnarled* trichome phenotype of Arabidopsis. Arabidopsis and soybean trichomes are morphologically distinct, with Arabidopsis exhibiting branched trichomes and soybean displaying unbranched trichomes. Despite such differences in form, mutations to the *nap1* gene in both species cause similar perturbations to trichome development and demonstrate that the two species share this key component in the actin polymerization pathway that underlies trichome morphology. This result suggests that other genes and pathways controlling trichome morphology are also likely to be conserved between the two species, which may facilitate the identification of other genes that are critical for soybean trichome function and development.

Several previous studies have described soybean trichome morphological mutants (Nagai and Saito 1923; Piper and Morse 1923; Stewart and Wentz 1926; Owen 1927; Johnson and Hollowell 1935; Ting 1946; Williams 1950; Bernard and Singh 1969; Bernard 1975; Healy et al. 2005). However, to our knowledge, no soybean study has cloned the underlying causative variant of a soybean trichome morphology mutant. The functional complementation of an Arabidopsis *nap1gnarled* mutant (*grl*-*4*) by whole plant transformation with *GmNAP1* validated the soybean *GmNAP1* gene function in the present study. Additionally, this study further validated the function of *GmNAP1* by identifying a second mutant allele of *GmNAP1*, the *p2* trichome mutant locus in line T31 (PI548159). This phenotype is the result of a single base pair deletion in the 22nd exon of *NAP1* (Fig. 5).

The T31 mutant is part of the USDA Soybean Isoline Collection and several previous studies have used mutants from this collection for trait mapping and candidate gene validation (Muehlbauer et al. 1991; Thompson et al. 1997; Cober and Voldeng 2001; Jeong et al. 2002; Molnar et al. 2003; Watanabe et al. 2009; Cober et al. 2010; Severin et al. 2010b; Peiffer et al. 2012). The recent genotyping of this collection with the SoySNP50K chip (Song et al. 2015) facilitated the rapid mapping of the T31 mutant. This study demonstrates the ability to leverage the valuable Isoline Collection historic mutant resource to validate a candidate gene. Efforts are currently underway to map additional mutant alleles from the Isoline Collection. To our knowledge, this is the first study to combine the SoySNP50K chip data with the Isoline Collection’s historic phenotypic data to validate a candidate gene by identifying a second mutant allele.

The 26.6 Mb mapping interval identified by WGS-BSA in this study was wider than expected, but the size of the interval was likely inflated by low regional recombination rates. The mapping interval had reasonable resolution on the distal side of the candidate gene with the mapping interval starting approximately 329 kb from *GmNAP1*. However, on the proximal side of *GmNAP1*, the mapping interval extended approximately 26.3 Mb from *GmNAP1* to the other arm of the chromosome. Due to the position of *GmNAP1* near the edge of the heterochromatic region, it was likely that repressed recombination on the proximal side of the gene expanded the mapping interval significantly. A recent mapping study also identified suppressed recombination in this region on chromosome 20 (Li et al. 2014). It is likely that the repressed recombination in this region led to the large mapping interval. Furthermore, it is probable that subsequent mapping studies using WGS-BSA in soybean will have smaller mapping intervals for regions of the genome with higher recombination rates.

Combining the aCGH data with the WGS-BSA mapping interval led to the identification of a single candidate gene, despite the large mapping interval. The only aCGH-detected mutation in the mapping interval was the approximately 2 kb deletion in Glyma.20G019300. Further examination at this locus identified additional mutations that were not detected by aCGH. These additional mutations include a second deletion, an inversion, and two novel junctions which suggest additional chromosome rearrangements occurred on chromosome 20. The complexity of fast neutron-induced mutations identified within this single gene was unexpected and further challenges the common assumption that fast neutron mutagenesis results in simple deletions (see Bolon et al. 2014 for additional evidence).

This study has demonstrated the effective combination of WGS-BSA and aCGH to identify a candidate fast neutron-induced mutation from a reasonable sized F_2_ mapping population. The combination of technologies demonstrated the ability to save significant cost and time by identifying the causative variant with only one round of BSA mapping, and without the need for additional fine-mapping.

### Author contribution statement

BWC, CPV, GJM, and RMS designed the research; BWC, ANH, SS, TJYK, FF, and JAO performed the research; BWC, TJYK and JAO analyzed the data; and BWC, JAO and RMS wrote the manuscript

## Electronic supplementary material

Below is the link to the electronic supplementary material.

**Supplemental Fig.** **1** The mutated Glyma.20G019300 allele co-segregates with the *gnarled* phenotype. **(a)** Three primers were used to generate a co-dominant marker that differentially amplifies wild-type and mutant alleles. The arrows indicate both the position and the direction of the primers B121R, B124R, and B124F. The B124F and B124R primers amplify a 708 bp fragment from the wild-type allele, and the B121R and B124R primers amplify a 188 bp fragment from the mutant allele. The combination of the inversion and deletion in the mutant allele orients the B124R primer such that it can amplify a fragment when paired with the B121R primer. **(b)** Perfect co-segregation was observed between the phenotypic classes and the expected genotypic classes among a population of 50 F_3_ individuals. The parent lines (R55C01 and ‘Noir 1’) are shown. Mutant (M) individuals exhibited only the 188 bp fragment, and wild-type (Wt) individuals exhibited either both fragments (heterozygous (Het)) or only the 708 bp fragment.**Supplemental Fig.** **2** RNA-seq read alignment density for each exon of Glyma.20G019300 in wild-type and *gnarled* mutant plants. RNA-seq reads mapping to Glyma.20G019300 clearly illustrate the lack of transcription from exons 12-14 in *NAP1* mutant plants. The height of the histogram indicates read depth along the length of the entire gene, with transcription peaks corresponding to exon sequences. Colored bars indicate SNPs relative to the ‘Williams 82’ reference genome sequence. Transcription of exon 11 appears to be up-regulated in mutant tissues compared to the wild-type. Exons 12, 13, and 14 are transcribed in all tissues of the wild-type plant but are not transcribed in the mutant plant, corresponding to the fast neutron induced deletions and structural rearrangements. Transcription of exons 15-23 is generally lower in tissues from the mutant plant compared to the wild-type plant**Supplemental Fig.** **3** Soybean *GmNAP1* functionally complements Arabidopsis *nap1* mutant (*grl*-*4*). **(a)** SEM image of wild-type trichomes on a Col-0 leaf. **(b)** SEM image of *gnarled* trichomes on a *nap1* mutant (*grl*-*4*). **(c)** SEM image of wild-type trichomes on a T_2_ *grl*-*4* plant complimented with the soybean *GmNAP1* transgene. Scale bars in **(a-c)** are each 200 um. **(d)** Leaf surface images of the Col-0, nap1 and T2 plants further confirmed successful complementation of this phenotype. **(e)** Amplification of *GmNAP1* transgene in 20 T_1_ Arabidopsis *grl*-*4* individuals with wild-type trichomes confirms that the *GmNAP1* is able to functionally compliment the Arabidopsis *nap1* mutant. From the left: soybean cv. ‘Williams 82’, Arabidopsis *nap1* mutant (*grl*-*4*), 20 Arabidopsis *grl*-*4* mutants transformed with the *GmNAP1* transgene and displaying a wild-type trichome phenotype. The fragment amplified spans from the promoter region into the first exon. The band size of ‘Williams 82’ is 548 bp, and the band size of the 20 Arabidopsis individuals is 556 bp. The difference of 8 bp is due to the insertion of an *AscI* restriction site in the *GmNAP1* transgene construct, just upstream of the ATG start site, which was added during construct assembly (PDF 1199 kb)
