# Supplementary material for: Fast neutron-induced structural rearrangements at a soybean NAP1 locus result in gnarled trichomes
Source: Theor Appl Genet. 2016 Jun 9;129:1725–38. doi: 10.1007/s00122-016-2735-x (PMC4983299; doi:10.1007/s00122-016-2735-x)
Supplement: Supplementary file 1 — Supplemental Fig. 1 The mutated Glyma.20G019300 allele co-segregates with the gnarled phenotype. (a) Three primers were used to generate a co-dominant marker that differentially amplifies wild-type and mutant alleles. The arrows indicate both the position and the direction of the primers B121R, B124R, and B124F. The B124F and B124R primers amplify a 708 bp fragment from the wild-type allele, and the B121R and B124R primers amplify a 188 bp fragment from the mutant allele. The combination of the inversion and deletion in the mutant allele orients the B124R primer such that it can amplify a fragment when paired with the B121R primer. (b) Perfect co-segregation was observed between the phenotypic classes and the expected genotypic classes among a population of 50 F3 individuals. The parent lines (R55C01 and ‘Noir 1’) are shown. Mutant (M) individuals exhibited only the 188 bp fragment, and wild-type (Wt) individuals exhibited either both fragments (heterozygous (Het)) or only the 708 bp fragment.Supplemental Fig. 2 RNA-seq read alignment density for each exon of Glyma.20G019300 in wild-type and gnarled mutant plants. RNA-seq reads mapping to Glyma.20G019300 clearly illustrate the lack of transcription from exons 12-14 in NAP1 mutant plants. The height of the histogram indicates read depth along the length of the entire gene, with transcription peaks corresponding to exon sequences. Colored bars indicate SNPs relative to the ‘Williams 82’ reference genome sequence. Transcription of exon 11 appears to be up-regulated in mutant tissues compared to the wild-type. Exons 12, 13, and 14 are transcribed in all tissues of the wild-type plant but are not transcribed in the mutant plant, corresponding to the fast neutron induced deletions and structural rearrangements. Transcription of exons 15-23 is generally lower in tissues from the mutant plant compared to the wild-type plantSupplemental Fig. 3 Soybean GmNAP1 functionally complements Arabidopsis nap1 mutant (grl-4). (a) SEM i [file 122_2016_2735_MOESM1_ESM.pdf]

List of Supplemental Tables

Supplemental Table 1: Codominant PCR primer triple used to amplify mutant and wild-type alleles

Supplemental Table 2: PCR Primers used to amplification across chromosome rearragements in Glyma.20G019300

Supplemental Table 3: PCR Primers used to sequence Glyma.20G019300 in line T31 (PI548159)

Supplemental Table 4: PCR Primers used to test for the presence of the *GmNAP1* construct in Arabidopsis T_1 individuals

Supplemental Table 1: Codominant PCR primer triple used to amplify mutant and wild-type alleles

Primer	Primer sequence	Description
B121R	TTTTCACCCTGTGGTTTTGG	Use Primer B121R with Primer B124R to amplify a 188 bp fragment across the mutant junction of Gm20:2,010,290 and Gm20:2,007,928
B124F	CGAGTGGTACCAGTGGACAT	Use Primer B124F with Primer B124R to amplify a 708 bp fragment in wild-type individuals
B124R	TGTCCCTTTGGCTTTAGT	Use Primer B124R with Primer B121R or Primer B124R to amplify either mutant or wild-type fragments, respectively

Supplemental Table 2: PCR Primers used to amplification across chromosome rearragements in Glyma.20G019300

Primer	Primer sequence	Description
B171F	GGTTGGGCATGAAGTGGTTC	Use B171 primer pair to amplify a 605bp fragment depicted in Fig 3F.
B171F	GATGTCCCACGCCGTTAAAG	Use B171 primer pair to amplify a 605bp fragment depicted in Fig 3F.
B172F	GAACCAACAGAGACTCGTGC	Use B172F and B173R primers to amplify a 1441bp fragment across the junction depicted in Fig 3E.
B173R	CCCTGTGGTTTTGGCAAGTT	Use B172F and B173R primers to amplify a 1441bp fragment across the junction depicted in Fig 3E.

Supplemental Table 3: PCR Primers used to sequence Glyma.20G019300 in line T31 (PI548159)

Primer	Primer sequence	Description
B230F	GCTTCATATTTCTCATTGAAAACC	Primers to amplify 5'UTR section of NAP1, 819bp, sequence with R primer.
B230R	CCACAGAACCAAATTCAAAGC	Primers to amplify 5'UTR section of NAP1, 819bp, sequence with R primer.
B244F	TTGTGGAGTTTGAGAAGCTTAGG	Primers to amplify exons: 1 & 2; sequence with both primers.
B244R	CTGGAATGTGAAAACCTTTGG	Primers to amplify exons: 1 & 2; sequence with both primers.
B231F	CATTCTCCTGCTATCATTGACC	Primers to amplify exons: 3,4,5; sequence with both primers.
B231R	GCTAGATTATACAACATGCTATGTCC	Primers to amplify exons: 3,4,5; sequence with both primers.
B232F	TTTTTCCCTAACCATTGTCACC	Primers to amplify exons: 6; sequence with F primer.
B232R	TGCTGCTCCTAAAAAGTAGAAAGG	Primers to amplify exons: 6; sequence with F primer.
B233F	TTCATTGTTTTCCTAGATTCTTTCC	Primers to amplify exons: 7,8; sequence with both primers.
B233R	TAAAACCAAACAATTTCAGTACCC	Primers to amplify exons: 7,8; sequence with both primers.
B234F	CCTTACTAATAGTCATCCAAATGTTGT	Primers to amplify exons: 9,10; sequence with one or both primers.
B234R	CCAAATCACTGAAAATAGCAACC	Primers to amplify exons: 9,10; sequence with one or both primers.
B235F	TGTTGATATTTGTTACTCTTTTCTGG	Primers to amplify exon: 11; sequence with F primer.
B235R	TGATATGAAACAACAAAAGGAGAGG	Primers to amplify exon: 11; sequence with F primer.
B236F	CTCTTCTGTGGACTCAGTGTGG	Primers to amplify exon: 12; sequence with F primer.
B236R	TCACCCCTTATGTTAGTTTTTGG	Primers to amplify exon: 12; sequence with F primer.
B237F	TGATGAATGGTTTGAAAAATGC	Primers to amplify exon: 13; sequence with F primer.
B237R	GAACGCATCTATTTGCATGG	Primers to amplify exon: 13; sequence with F primer.
B238F	TTTTGCATGGGTGTTTTGG	Primers to amplify exons: 14,15; sequence with both primers.
B238R	GATCATGATTTTGACTATACCATCG	Primers to amplify exons: 14,15; sequence with both primers.
B239F	TGTAACAGCTGAGTTAGAGCTTCC	Primers to amplify exons: 16,17; sequence with both primers.
B239R	ATGCCTCCATCAAAATGTGC	Primers to amplify exons: 16,17; sequence with both primers.
B240F	TGTGTGATGGTAGCAATATGTGG	Primers to amplify exons: 18,19,20; sequence with both primers.
B240R	TTTCTTTAAAGGGCGATACCC	Primers to amplify exons: 18,19,20; sequence with both primers.
B241F1	TCCAGATCCAACATTAGTCACC	Primers to amplify exon: 21; Amplify with F1, R. sequence with all 3 primers.
B241F2	AGGACCGGTTTCTTCTCTGC	Primers to amplify exon: 21; Amplify with F1, R. sequence with all 3 primers.

B241R	CAATGGCAATGAATAGTTCAGC	Primers to amplify exon: 21; Amplify with F1, R. sequence with all 3 primers.
B242F	GCTGAACTATTCATTGCCATTG	Primers to amplify exon: 22; sequence with F primers.
B242R	AATATATTGCAACATTGCCTACC	Primers to amplify exon: 22; sequence with F primers.
B243F1	GAATTTCACCTGCGGTTTTG	Primers to amplify exon: 23 and 3'UTR; Amplify with F1, R. sequence with all 3 primers.
B243F2	TATTATGGGCACGAATCAGG	Primers to amplify exon: 23 and 3'UTR; Amplify with F1, R. sequence with all 3 primers.
B243R	ACTTGTACTCGAGCGGCATT	Primers to amplify exon: 23 and 3'UTR; Amplify with F1, R. sequence with all 3 primers.

Supplemental Table 4: PCR Primers used to test for the presence of the *GmNAP1* construct in Arabidopsis T_1 individuals

Primer	Primer sequence	Description
B182F	ATTCGTGCTTACAACTCGCC	Use primers B182F and B182R to amplify a 548 bp band from Soybean plants or a 556 bp band from Arabidopsis plants with the *GmNAP1* construct.
B182R	CCTGAGACTGCCCATCATGA	Use primers B182F and B182R to amplify a 548 bp band from Soybean plants or a 556 bp band from Arabidopsis plants with the *GmNAP1* construct.

List of Supplemental Figures

Supplemental Fig. 1: The mutated Glyma.20G019300 allele co-segregates with the gnarled phenotype.

Supplemental Fig. 2: RNA-seq read alignment density for each exon of Glyma.20G019300 in wild-type and gnarled mutant plants.

Supplemental Fig. 3: Soybean *GmNAP1* functionally complements Arabidopsis *nap1* mutant (*grl-4*).

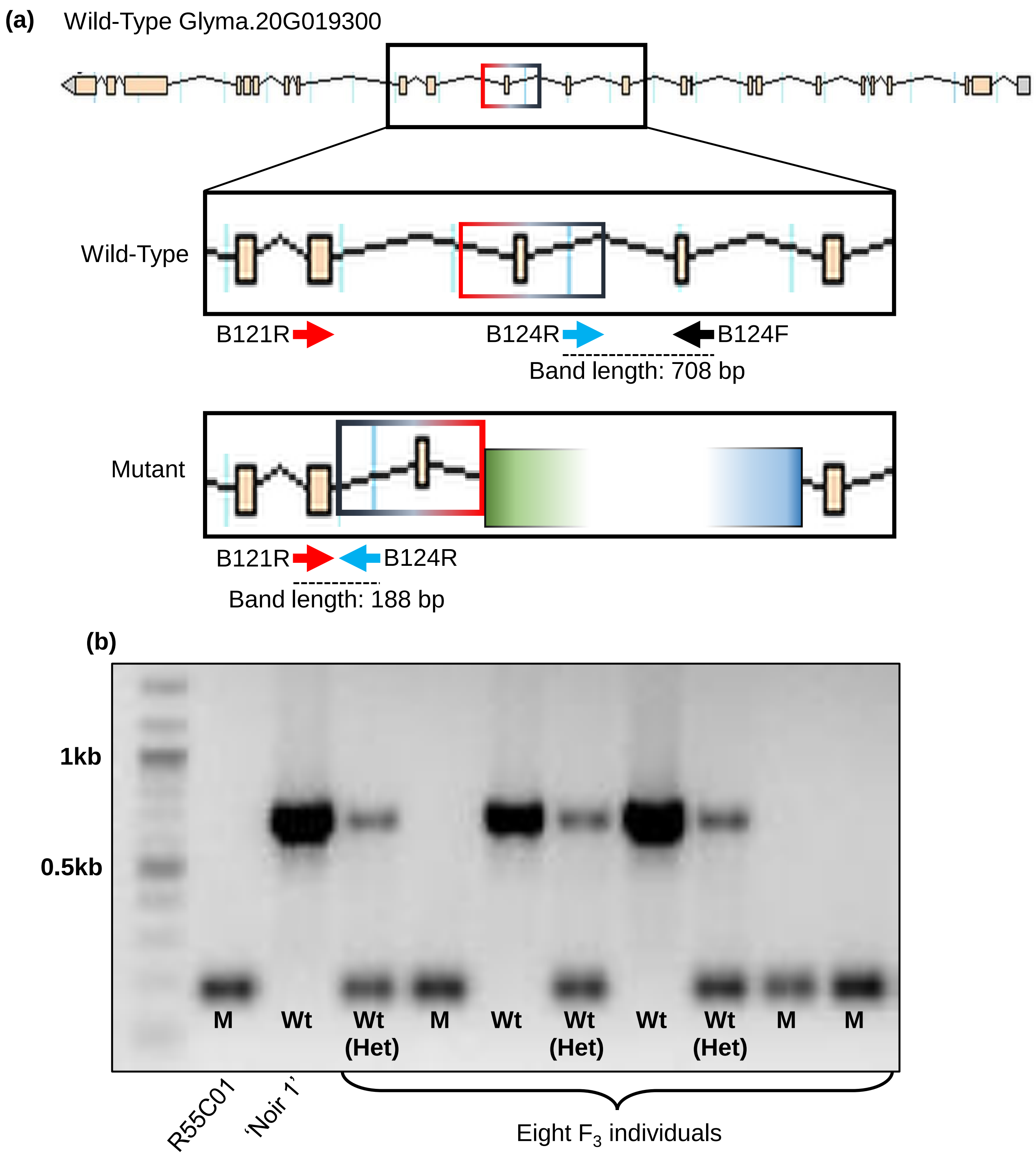

Supplemental Fig. 1 The mutated Glyma.20G019300 allele co-segregates with the *gnarled* phenotype. **(a)** Three primers were used to generate a co-dominant marker that differentially amplifies wild-type and mutant alleles. The arrows indicate both the position and the direction of the primers B121R, B124R, and B124F. The B124F and B124R primers amplify a 708 bp fragment from the wild-type allele, and the B121R and B124R primers amplify a 188bp fragment from the mutant allele. The combination of the inversion and deletion in the mutant allele orients the B124R primer such that it can amplify a fragment when paired with the B121R primer. **(b)** Perfect co-segregation was observed between the phenotypic classes and the expected genotypic classes among a population of 50 F_3 individuals. The parent lines (R55C01 and 'Noir 1') are shown. Mutant (M) individuals exhibited only the 188 bp fragment, and wild-type (Wt) individuals exhibited either both fragments (heterozygous (Het)) or only the 708 bp fragment.

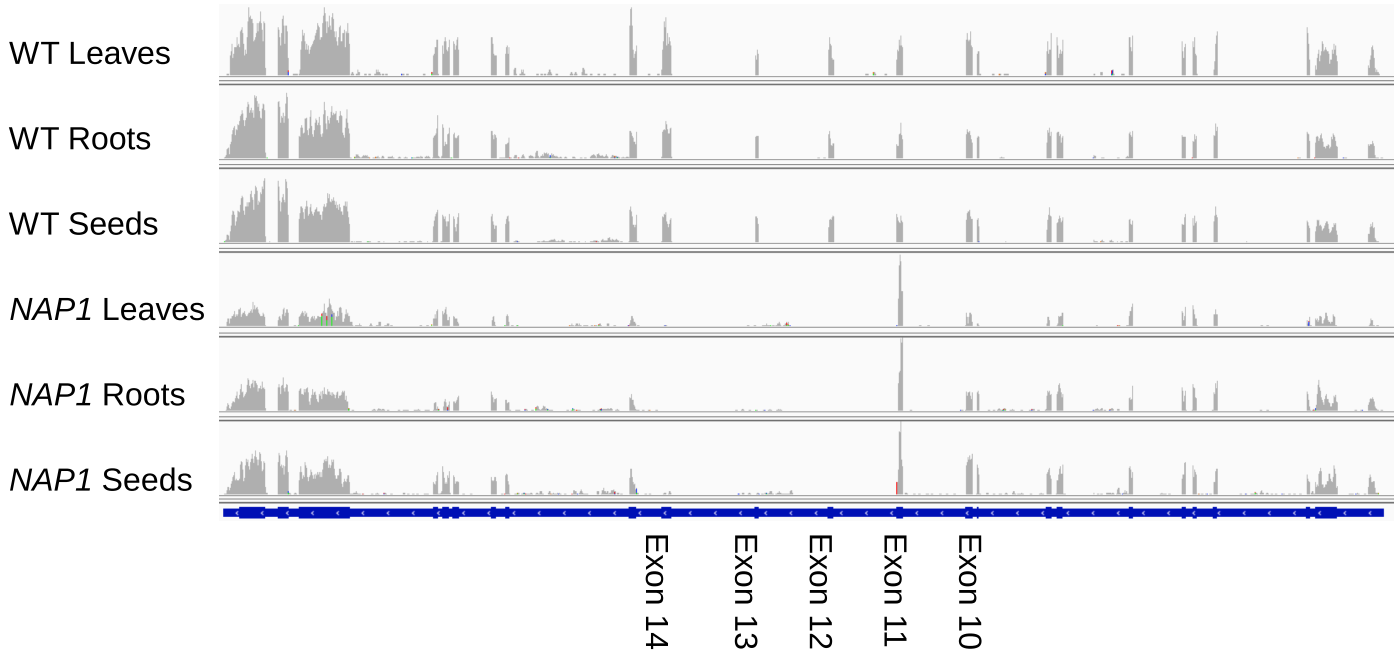

Supplemental Fig. 2 RNA-seq read alignment density for each exon of Glyma.20G019300 in wild-type and *gnarled* mutant plants. RNA-seq reads mapping to Glyma.20G019300 clearly illustrate the lack of transcription from exons 12-14 in *NAP1* mutant plants. The height of the histogram indicates read depth along the length of the entire gene, with transcription peaks corresponding to exon sequences. Colored bars indicate SNPs relative to the 'Williams 82' reference genome sequence. Transcription of exon 11 is statistically up-regulated in mutant tissues compared to the wild-type. Exons 12, 13, and 14 are transcribed in all tissues of the wild-type plant but are not transcribed in the mutant plant, corresponding to the fast neutron induced deletions and structural rearrangements. Transcription of exons 15-23 is generally lower in tissues from the mutant plant compared to the wild-type plant.

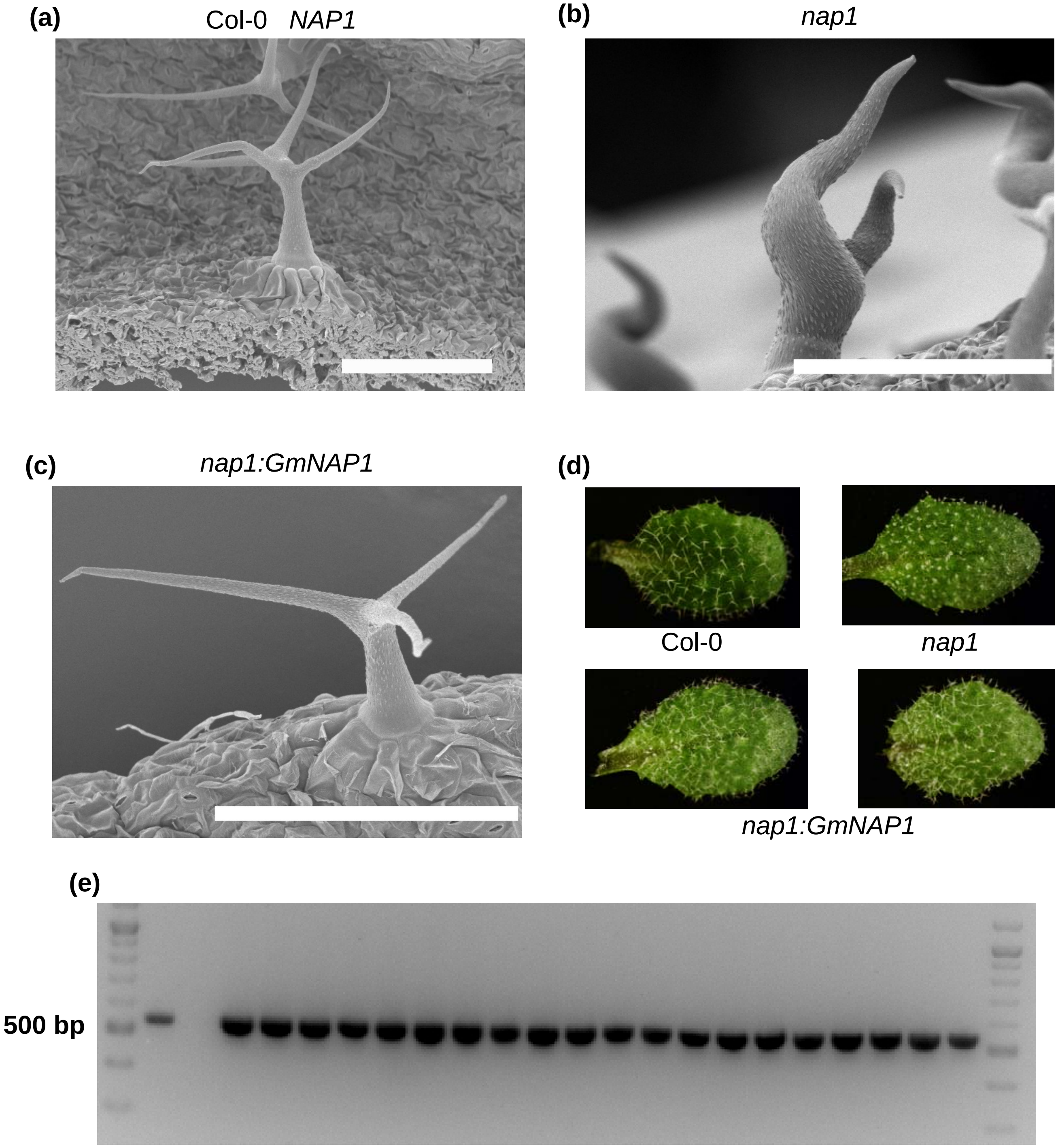

Supplemental Fig. 3 Soybean *GmNAP1* functionally complements Arabidopsis *nap1* mutant (*grl-4*). **(a)** SEM image of wild-type trichomes on a Col-0 leaf. **(b)** SEM image of *gnarled* trichomes on a *nap1* mutant (*grl-4*). **(c)** SEM image of wild-type trichomes on a T_2 *grl-4* plant complemented with the soybean *GmNAP1* transgene. Scale bars in **(a-c)** are each 200 um. **(d)** Leaf surface images of the Col-0, *nap1* and T_2 plants further confirmed successful complementation of this phenotype. **(e)** Amplification of *GmNAP1* transgene in 20 T_1 Arabidopsis *grl-4* individuals with wild-type trichomes confirms that the *GmNAP1* is able to functionally compliment the Arabidopsis *nap1* mutant. From the left: soybean cv. 'Williams 82', Arabidopsis *nap1* mutant (*grl-4*), 20 Arabidopsis *grl-4* mutants transformed with the *GmNAP1* transgene and displaying a wild-type trichome phenotype. The fragment amplified spans from the promoter region into the first exon. The band size of 'Williams 82' is 548 bp, and the band size of the 20 Arabidopsis individuals is 556 bp. The difference of 8 bp is due to the insertion of an *AscI* restriction site in the *GmNAP1* transgene construct, just upstream of the ATG start site, which was added during construct assembly.
